# Abnormal frequency of the memory B cell subsets and plasmablasts in patients with congenital severe hemophilia A: correlation with “Inhibitor” formation

**DOI:** 10.1007/s44313-024-00017-7

**Published:** 2024-04-16

**Authors:** Omid Reza Zekavat, Yasaman Movahednezhad, Amin Shahsavani, Sezaneh Haghpanah, Negin Shokrgozar, Hossein Golmoghaddam, Mehdi Kalani, Mohammad Reza Bordbar, Nargess Arandi

**Affiliations:** 1 Hematology Research Center, Shiraz University of Medical Sciences, Shiraz, Iran; 2 Department of Pathology, School of Medicine, Shiraz University of Medical Sciences, Shiraz, Iran; 3 Department of Immunology, Professor Alborzi Clinical Microbiology Research Center, Shiraz University of Medical Sciences, Shiraz, Iran

**Keywords:** Severe hemophilia A, Inhibitors, Memory B cells, Plasmablasts

## Abstract

**Background:**

Development of antibodies against infused Factor VIII (FVIII) or "inhibitors" represents a major challenge following FVIII replacement therapy in patients with hemophilia A (HA). Recent studies have shown that certain cellular compartments of the immune system contribute to the production of such antibodies. Herein, we determined the frequency of class-switched CD19^+^IgD^−^CD27^+^/non-class-switched CD19^+^IgD^+^CD27^+^ memory B cell subsets and CD19^+^CD27^hi^CD38^hi^ plasmablasts in patients with severe HA and their association with the development of inhibitors in these patients.

**Methods:**

This cross-sectional case–control study enrolled 32 patients with severe HA, including 8 with and 24 without inhibitors, and 24 healthy individuals. The frequencies of the memory B cell subsets and plasmablasts were determined using flow cytometry.

**Results:**

The frequency of CD19^+^IgD^+^CD27^+^ non-class-switched memory B cells was significantly lower in patients with HA (including both patients with and without inhibitors) than in healthy controls. The percentages of both CD19^+^IgD^−^CD27^+^ class-switched and CD19^+^IgD^+^CD27^+^ non-class-switched memory B cells did not differ significantly between patients with and without inhibitors. HA patients with inhibitors had significantly higher proportions of CD19^+^CD27^hi^CD38^hi^ plasmablasts than the control group as well as the inhibitor (-) ones. No significant correlation was observed between the inhibitor levels with the percentages of memory B cell subsets and plasmablasts.

**Conclusion:**

This study is the first to demonstrate a dysregulated proportion of CD19^+^IgD^+^CD27^+^ non-class-switched memory B cells and CD19^+^CD27^hi^CD38^hi^ plasmablasts in patients with severe HA. Therefore, strategies targeting memory B-cell/plasmablast differentiation may have promising outcomes in the management of inhibitor formation in patients with severe HA.

## Introduction

The development of antibodies against coagulation factor VIII (anti-FVIII), known as "inhibitors," represents a major complication following replacement therapy with exogenous FVIII in patients with hemophilia A (HA) [1–5]. These antibodies can effectively neutralize the biological activity of the administered FVIII, significantly impairing the efficacy of the replacement therapy [1–5]. The mechanisms underlying inhibitor production in some patients remain unclear. However, recent studies have suggested that single nucleotide polymorphisms in immune response genes and alterations in the plasma levels of specific cytokines are linked to inhibitor formation in HA patients [6–8]. Furthermore, a growing body of evidence implicates the role of certain cellular compartments of the adaptive immune system, such as regulatory T cells, in the development of inhibitors in these patients [9–12].

B-lymphocytes are a major component of the humoral immune responses. Upon encountering specific cognate antigens, they are activated, leading to their differentiation into memory B cells and plasmablasts [13, 14]. While memory B cells contribute to maintaining immunological memory responses, plasmablasts are responsible for antibody production following their development into plasma cells in the bone marrow, thereby serving as the first line of defense upon re-exposure to the same antigens [13–15].

For several years, the CD27 receptor has been used as a unique marker to distinguish memory cells from naïve B cells. However, recent advances in memory B-cell classification have identified several subtypes with distinct functions. Among these subtypes, IgD^+^ memory B cells (non-class-switched) and IgD^−^ memory B cells (class-switched) have received increased attention [13–16].

Limited data are available regarding the role of memory B cell subsets in the development of inhibitors in patients with HA [17], and no information is currently available regarding the proportion of plasmablasts in these patients.

Consequently, this study is the first to investigate the frequency of memory B cell subtypes, including class-switched and non-class-switched memory B cells, as well as plasmablasts, in patients with severe HA. Additionally, we explored the potential correlation between these subsets and the development of inhibitors in patients with severe HA.

### Patients and methods

#### Patients’ selection criteria

This was a cross-sectional, case–control study involving 32 male patients with severe HA who were referred to the Comprehensive Hemophilia Center affiliated with Shiraz University of Medical Sciences between January and December 2021. Among these 32 patients with HA, eight were positive for inhibitors at the time of recruitment, while the remaining 24 patients were negative for inhibitors, which were defined as the inhibitor ( +) and inhibitor (-) groups, respectively. The healthy control group consisted of 24 healthy males with no history of infectious, inflammatory, or malignant diseases.

The exclusion criteria included a positive history of collagen vascular or chronic infectious diseases; positive serological results for hepatitis B or C; bacterial infectious diseases within the past 2 months; a history of consuming immunosuppressive drugs, such as glucocorticosteroids and cyclosporine; and/or confirmed autoimmune, inflammatory, or allergic disorders. All patients included in this study had chronic hemophilic arthropathy and no history of immune tolerance induction.

The study protocol was approved by the Institutional Ethics Committee of Shiraz University of Medical Sciences (IR.SUMS.MED.REC.1398.605), and all enrolled patients and/or their guardians provided written informed consent.

#### Factor VIII activity and inhibitor titration

Factor VIII activity levels were determined as previously described [9]. Inhibitor titration was performed using the Nijmegen-Bethesda inhibitory assay to determine the inhibitory antibodies against Factor VIII [18]. Patients with the inhibitor levels > 0.6 BU/mL measured using the Bethesda assay were defined as inhibitor-positive individuals.

#### Sample collection

Five milliliters of the peripheral blood specimens were obtained and collected from all patients and controls in the ethylenediaminetetraacetic acid (EDTA)-containing tubes. The peripheral blood mononuclear cells were isolated using Ficoll-hypaque reagent (Inno-Train, Germany) via density gradient centrifugation at 400 × g for 20 min. Subsequently, the cells were washed with phosphate-buffered saline (PBS) prior to flow cytometry staining.

#### Antibodies

Memory B cells and plasmablasts were stained with the following monoclonal antibodies: PE/Cy5 anti-human CD19 (Clone HIB19), FITC anti-human CD27 (Clone M-T271), PE anti-human IgD (Clone IA6-2), and APC anti-human CD38 (Clone HIT2). All antibodies were purchased from BioLegend Company (USA).

#### Flow cytometry staining of memory B cells and plasmablasts

The isolated mononuclear cells were resuspended in 100 µL of staining buffer and incubated with antibodies according to the manufacture’s recommendation. A three-color flow cytometry staining method was used.

Flow cytometric analysis of the stained cells was performed using a FACSCalibur flow cytometer (BD Biosciences), and the flow cytometry data were analyzed using FlowJo software version 7.6. The following gating strategy was used to enumerate memory B cells and plasmablast population.

**Fig. 1:**
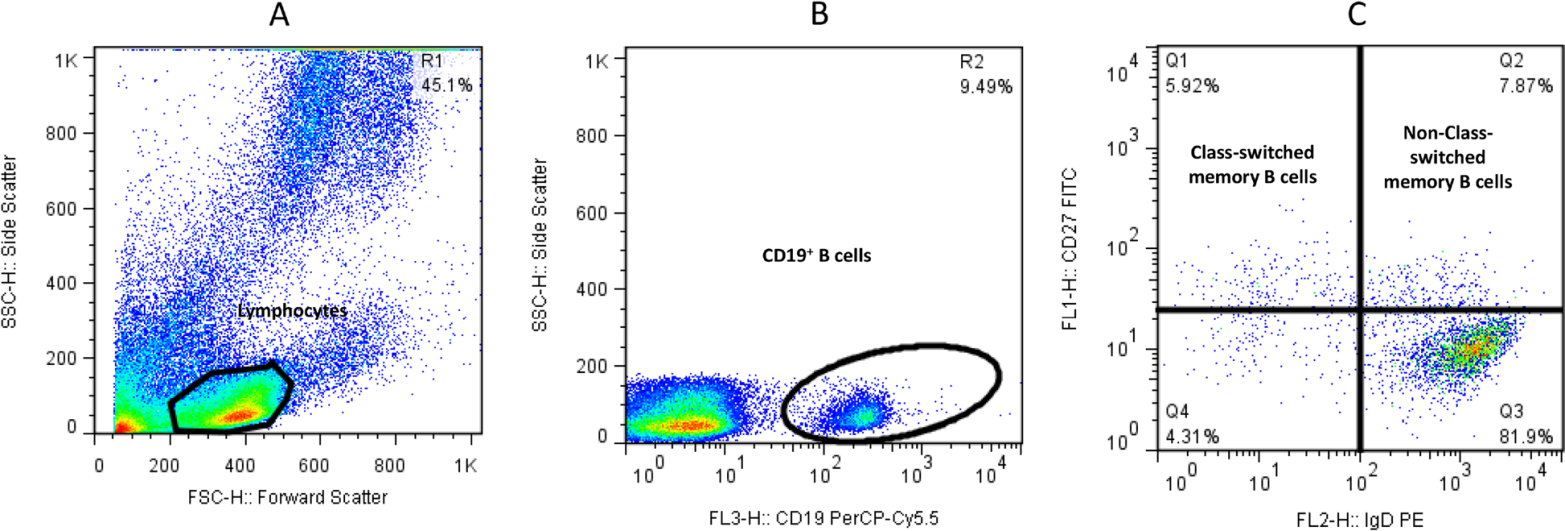
Flow cytometry analysis of class-switched (CD19^+^IgD^−^CD27^+^) and non-class switched (CD19^+^IgD^+^CD27^+^) memory B cells

**Fig. 2:**
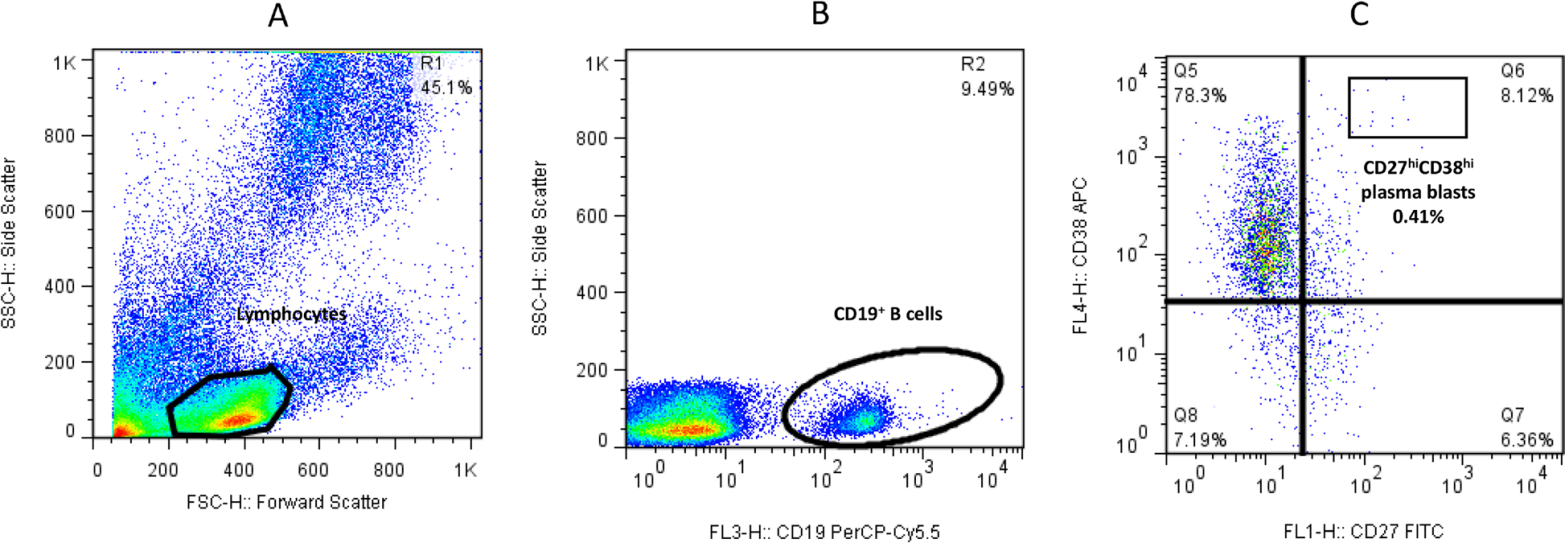
Flow cytometry analysis of CD19^+^CD27^hi^CD38^hi^ plasmablasts

First, the lymphocyte population was gated based on the forward *vs.* side scatter plot (A). Second, the population of CD19^+^ B cells was selected from the lymphocyte gates (B). Third, the population of IgD^+^CD27^+^ and IgD^−^CD27^+^ cells within the CD19^+^ B cells was defined as non-class switched CD19^+^IgD^+^CD27^+^ (Q2) and class- switched CD19^+^IgD^−^CD27^+^ memory B cells (Q1), respectively (C) (Fig. 1). For plasmablasts, after gating CD19^+^ B cells among the lymphocyte population (B), cells were defined based on CD27 vs. CD38 expression (C), and the CD27^+^CD38^+^ population (Q6) that expressed high levels of both markers was recognized as CD19^+^CD27^hi^CD38^hi^ plasmablasts (Fig. 2).

#### Statistical analysis

Statistical analysis was conducted using the Statistical Package for the Social Sciences version 22.0. Quantitative variables were presented as mean ± standard deviation (SD). To compare quantitative variables between the two groups with parametric and non-parametric distributions, Student’s t-test and Mann–Whitney test were employed, respectively. For comparisons involving more than two groups, Kruskal–Wallis tests were used. The Spearman's correlation test was used to assess the associations between quantitative variables. Statistical significance was set at *P* < 0.05.

## Results

In this study, 32 male patients with severe HA were included, comprising 8 with inhibitors (inhibitor +) and 24 without inhibitors (inhibitor-), along with 24 healthy male volunteers. The mean age of patients with HA and controls was 23.84 ± 15.89 (range 2–50 years) and 25.23 ± 14.46 (range 3–52 years), respectively. The clinical and laboratory data for patients with HA are presented in Table 1.

**Table 1:** Laboratory and clinical characteristics of patients with HA

Variables	Inhibitor ( +) ( *n* = 8)	Inhibitor (-) ( *n* = 24)
WBC count (× 10^3^/µL)	6.23 ± 0.95	6.07 ± 0.32
Platelets (× 10^3^/µL)	323.33 ± 63.72	250.62 ± 16.9
Hb (g/dL)	11.92 ± 1.02	14.12 ± 0.39
FVIII concentration (IU/mL)	0.61 ± 0.14	2.2 ± 0.63
Anti-FVIII inhibitor titer (BU/mL)	6.15 ± 2.8	< 0.6
Received therapy		
pFVIII	6	6
rFVIII	0	18
Unknown	2	0
Type of treatment		
On-demand	3	16
Prophylaxis	4	7
Unknown	1	1
Chronic hemophilic arthropathy	8 (100%)	24 (100%)
Previous history of viral hepatitis		
Positive	0	2
Negative	6	11
Suspicious	1	10
Unknown	1	1

Results are shown as mean ± SD

*WBC* White blood cell, *Hb* Hemoglobin, *pFVIII* Plasma-derived FVIII, *rFVIII* Recombinant FVIII

### Frequency of memory B cells and plasmablasts in HA patients and healthy controls

The frequencies of class-switched CD19^+^IgD^−^CD27^+^ and non-class-switched CD19^+^IgD^+^CD27^+^ memory B cells, as well as CD19^+^CD27^hi^CD38^hi^ plasmablasts, were determined and compared between patients with HA and healthy controls (Table 2).

**Table 2:** Frequencies of memory B cell subsets and plasmablasts in patients with severe HA and controls

Parameters	Patients with HA (*n* = 32)	Controls (*n* = 24)	*P*-value
Class-switched memory B cells (%) (CD19 + IgD − CD27 + )	10.39 ± 4.74	13.97 ± 8.93	0.089
Non-class switched memory B cells (%) (CD19^+^IgD^+^CD27^+^)	7.53 ± 4.03	11.16 ± 6.74	*0.016
Plasmablasts (%) (CD19 ^+^CD27 ^hi^CD38 ^hi^)	0.33 ± 0.04	0.1 ± 0.02	* < 0.001

Data are presented as mean ± SD

**Fig. 3:**
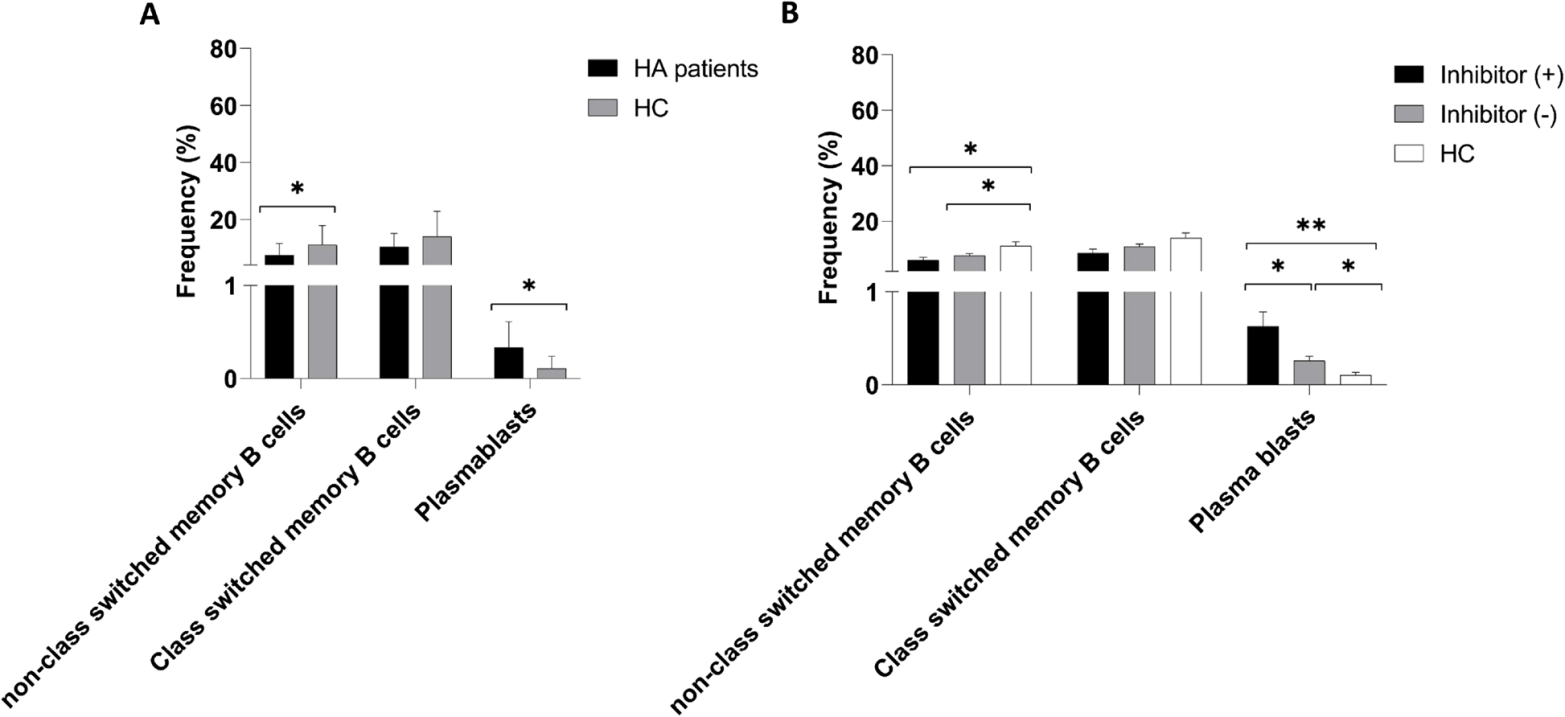
Comparison of the frequency of memory B cell subsets and plasmablasts between patients with HA and healthy controls (**A**) and between HA patients with/without inhibitors and healthy controls (**B**) Data are presented as mean ± SD; *P < 0.05, **P < 0.001

Analysis revealed that patients with HA had a significantly lower number of non-class-switched CD19^+^IgD^+^CD27^+^ memory B cells than the healthy control group (7.53 ± 4.03% vs*.* 11.16 ± 6.74%; *P = 0.016) (Fig. 3). Although the frequency of class-switched CD19^+^IgD^−^CD27^+^ memory B cells was lower in patients with HA than that in the control group, the difference was not statistically significant (10.39 ± 4.74% vs*.* 13.97 ± 8.93%; *P* = 0.089) (Fig. 3).

Interestingly, patients with HA had a significantly higher number of CD19^+^CD27^hi^CD38^hi^ plasmablasts than the healthy control group (0.34 ± 0.06% vs*.* 0.1 ± 0.02%; *P < 0.001) (Fig. 3).

### Comparison of the frequency of memory B cells and plasmablasts between HA patients with/without inhibitors and healthy controls

The frequencies of class-switched CD19^+^IgD^−^CD27^+^ memory B cells, non-class-switched CD19^+^IgD^+^CD27^+^ memory B cells, and CD19^+^CD27^hi^CD38^hi^ plasmablasts were determined and compared between HA patients with and without inhibitors and healthy controls (Table 3).

**Table 3:** Frequency of memory B cell subsets and plasmablasts in HA patients with ( +) and without (-) inhibitors and controls

Parameters	Inhibitor ( +) (*n* = 8)	Inhibitor (-) ( n = 24)	Controls (*n* = 24)	P1	P2	P3
Class-switched memory B cells (%) (CD19^+^IgD^−^CD27^+^)	8.84 ± 4.64	10.9 ± 4.76	13.97 ± 8.93	0.22	0.172	0.153
Non-class switched memory B cells (%) (CD19^+^IgD^+^CD27^+^)	7.12 ± 5.58	7.67 ± 3.51	11.16 ± 6.74	0.4	*0.04	*0.034
Plasmablasts (%) (CD19 ^+^CD27 ^hi^CD38 ^hi^)	0.55 ± 0.09	0.25 ± 0.05	0.1 ± 0.02	*0.003	* < 0.001	*0.013

Data are presented as mean ± SD

P1: Comparison of parameters between inhibitor ( +) vs*.* inhibitor ( +)

P2: Comparison of parameters between the inhibitor ( +) vs. control groups

P3: Comparison of parameters between inhibitor (-) vs*.* controls

The results indicated no significant difference in the percentage of non-class-switched CD19^+^IgD^+^CD27^+^ and class-switched CD19^+^IgD^−^CD27^+^ memory B cells between inhibitor ( +) and (-) patients (7.12 ± 5.58% vs*.* 7.67 ± 3.51%; *P* = 0.4 and 8.84 ± 4.64% vs. 10.9 ± 4.76%; *P* = 0.22, respectively) (Fig. 3). However, the percentages of non-class-switched CD19^+^IgD^+^CD27^+^ memory B cells were significantly lower in both inhibitor ( +) and inhibitor (-) patients with HA than that in the healthy controls (7.12 ± 5.58% vs*.* 11.16 ± 6.74%; *P = 0.04 and 7.67 ± 3.51% vs*.* 11.16 ± 6.74%; *P = 0.034) (Fig. 3). The frequency of class-switched CD19^+^IgD^−^CD27^+^ memory B cells did not differ significantly among the three groups (*P* > 0.05) (Fig. 3).

In contrast, the frequency of CD19^+^CD27^hi^CD38^hi^ plasmablasts was higher in patients with HA than that in the control group (0.33 ± 0.04% vs*.* 0.1 ± 0.02%; *P < 0.001) (Fig. 3).

Furthermore, CD19^+^CD27^hi^CD38^hi^ plasmablasts were significantly more frequent in HA patients with inhibitors (inhibitor +) than those in both inhibitor (-) patients and healthy controls (0.55 ± 0.09% vs*.* 0.25 ± 0.05%; *P = 0.003 and 0.55 ± 0.09% vs. 0.1 ± 0.02%; *P < 0.001, respectively) (Fig. 3). Additionally, HA patients without inhibitors had significantly higher CD19^+^CD27^hi^CD38^hi^ plasmablasts than the control group (0.25 ± 0.05% vs*.* 0.1 ± 0.02%; *P = 0.013) (Fig. 3).

### Correlation among memory B cells, plasmablasts, and inhibitor titers

**Fig. 4:**
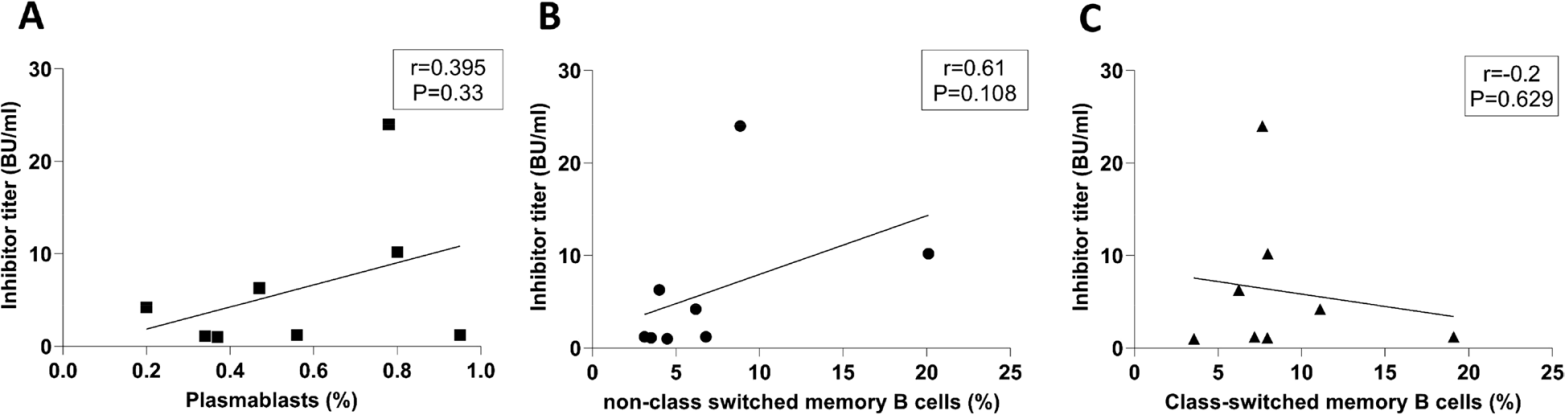
Correlation between the inhibitor levels with the frequency of class-switched CD19^+^CD27^+^IgD^−^ memory B cells (**A**), non-class-switched CD19^+^CD27^+^IgD^+^ memory B cells (**B**) and plasmablasts (**C**) in inhibitor ( +) patients with HA

The correlation among the frequency of memory B cell subsets, plasmablasts, and inhibitor titers was evaluated in inhibitor (+) patients. The results revealed no significant correlation between the inhibitor levels with the frequency of class-switched, non-class-switched memory B cells and CD19^+^CD27^hi^CD38^hi^ plasmablasts (*P* > 0.05) (Fig. 4).

## Discussion

It is well established that the immune system plays a pivotal role in the development of inhibitors in patients with HA. In line with this, previous studies have indicated the role of a specific subset of T cells, including regulatory T cells (Tregs) in the formation of inhibitors in patients with severe HA [9–12]. However, data on the contribution of B cell subpopulations, such as memory B cells and plasmablasts in this context remain limited.

In this study, we assessed the frequencies of memory B cell subtypes (both class-switched and non-class-switched memory B cells) and plasmablasts to determine whether they were associated with inhibitor formation in patients with severe HA.

Our results demonstrated that the percentages of both class-switched and non-class-switched memory B cells did not differ between HA patients with and without inhibitors. However, the percentages of non-class-switched CD19^+^IgD^+^CD27^+^ memory B cells were significantly lower in patients with HA, including both inhibitor ( +) and inhibitor (-) patients, than that in healthy controls. The frequency of class-switched CD19^+^IgD^−^CD27^+^ memory B cells did not differ significantly among the three groups.

Notably, HA patients, particularly those with inhibitors, had significantly elevated levels of CD19^+^CD27^hi^CD38^hi^ plasmablasts in their peripheral blood compared to both inhibitor (-) patients and healthy controls.

The immunological memory of B cells that is produced following re-exposure to the same antigens is often elicited by T cell-dependent antigens and is characterized by rapid humoral immune responses of greater magnitude, containing antibodies with higher affinity (compared to the primary immune responses) [13, 16]. Recent advances have revealed heterogeneity in human memory B cells, which have introduced two major subtypes based on IgD/IgM expression; class-switched and non-class-switched memory B cells [13, 16]. The molecular mechanisms underlying the differentiation of these two populations remain unknown, but they likely have distinct functional properties. While the IgD^+^/IgM^+^ non-class switched memory B cells are responsible for replenishment of the memory B cell pool by their rapid localization into germinal center (GC) loci, the IgD^−^ class-switched memory B cells rapidly differentiate into class-witched plasma cells [13, 16]. Moreover, there are evidences showing that the non-class-switched IgD^+^/IgM^+^ memory B cells can be produced during T cell-independent (TI) humoral immune responses outside germinal centers [13, 16]. Interestingly, a recent report by Patel et al. explained that both GC-dependent and -independent mechanisms may be evoked by frequent FVIII infusions in patients with HA [19].

The clinical significance of the low percentages of the non-class switched memory B cells in patients with HA, including both inhibitor (+) and inhibitor (-) ones, in our study remains elusive. However, as this memory B cell subpopulation has been considered responsible for the preservation of the memory B cell pool, our findings may reveal dysregulated memory B cell maintenance/renewal in patients with and without inhibitors. Another hypothesis is that non-class-switched memory B cells are produced by eliciting TI immune responses toward FVIII in patients with HA; however, they may diminish owing to their differentiation into plasma cells following repeated re-exposure to infused FVIII. Further research is required to verify these assumptions.

Plasmablasts are terminally differentiated B cells that migrate from germinal centers into circulation and eventually differentiate into plasma cells in the bone marrow [20]. These rapidly produced plasmablasts are short-lived effector cells that are responsible for early antibody responses, whereas long-lived plasma cells sustain durable humoral immunity [20]. To date, there have been no reports on the percentage of plasmablasts in patients with HA .

The significantly higher proportion of plasmablasts observed in our inhibitor (+) patients may explain why they were reactivated due to repeated exposure to external FVIII, potentially contributing to the high levels of inhibitor formation in these patients.

Our results suggest that in addition to immune tolerance induction (ITI) treatment, a widely accepted therapeutic approach to reduce inhibitor levels and induce immune tolerance in patients with HA [21, 22], drugs targeting memory B cells and/or plasmablasts/plasma cell differentiation might have promising results in reducing inhibitor levels, thus alleviating the clinical symptoms related to inhibitor formation in HA patients. In this regard, administration of a blocking antibody that targets chemokine/chemokine receptors responsible for the homing of plasmablasts into the bone marrow to produce antibody-secreting plasma cells, as well as the transcription factors involved in plasmablast/plasma cell differentiation, might be helpful [23, 24]. Moreover, a recent study by Doshi et al. revealed that soluble factors/receptors that affect B cell maturation and survival, including B cell–activating factor (BAFF),  a proliferation-inducing ligand (APRIL), and B cell maturation antigen (BCMA), may be implicated in the development of inhibitors for patients with HA thus may represent potential therapeutic targets [25]. They introduced BAFF as a new modulator for inhibitor formation in HA patients that can be a suitable target in conjunction with α-CD20 therapy for eradication of the FVIII-specific inhibitors these  patients using anti-BAFF antibody (Belimumab) [25].

Blocking the co-stimulatory interactions between memory B cells and activated T cells/dendritic cells required for re-stimulation of FVIII-specific memory B cells may prevent FVIII-specific memory-B-cell reactivation [26]. Therefore, it can be considered as a suitable candidate therapeutic strategy to interfere with and/or avoid inhibitor formation in patients with HA.

Our study also revealed no significant correlation between the inhibitor levels with the percentages of class-switched memory B cells, non-class-switched memory B cells, and CD19^+^CD27^hi^CD38^hi^ plasmablastsin inhibitor (+) patients. The limited number of inhibitor (+) patients in our study may have contributed to this lack of significance. Therefore, larger multicenter studies are recommended to validate our results. Additionally, investigating the dynamic changes in memory B cell subtypes and plasmablasts during ITI in a sufficient number of patients with HA could provide valuable insights into their roles in controlling inhibitor formation. Characterizing FVIII-specific memory B cell populations in the peripheral blood may further elucidate their implications for inhibitor production in patients with HA.

## Conclusion

To the best of our knowledge, our study provides the first evidence of an altered frequency of non-class-switched CD19^+^IgD^+^CD27^+^ memory B cells and CD19^+^CD27^hi^CD38^hi^ plasmablasts in patients with HA. Therefore, strategies targeting memory B cell/plasmablast differentiation might have promising outcomes in the management of inhibitor formation in patients with severe HA. Further research is needed to fully understand the mechanisms underlying these findings and to explore novel treatment strategies targeting these immune components.

## Data Availability

The datasets generated and/or analyzed during the current study are available from the corresponding author upon reasonable request.
