# Correction: Abnormal frequency of the memory B cell subsets and plasmablasts in patients with congenital severe hemophilia A: correlation with “Inhibitor” formation

**DOI:** 10.1007/s44313-024-00019-5

**Published:** 2024-04-26

**Authors:** Omid Reza Zekavat, Yasaman Movahednezhad, Amin Shahsavani, Sezaneh Haghpanah, Negin Shokrgozar, Hossein Golmoghaddam, Mehdi Kalani, Mohammad Reza Bordbar, Nargess Arandi

**Affiliations:** 1 Hematology Research Center, Shiraz University of Medical Sciences, Shiraz, Iran; 2 Department of Pathology, School of Medicine, Shiraz University of Medical Sciences, Shiraz, Iran; 3 Department of Immunology, Professor Alborzi Clinical Microbiology Research Center, Shiraz University of Medical Sciences, Shiraz, Iran

**Correction: Blood Res 59, 16 (2024)**

**https://doi.org/10.1007/s44313-024-00017-7**

In this article [1], the received date was inadvertently omitted and should have been 22 November 2023.

The original article has been corrected.

## References

[1] Zekavat OR, Movahednezhad Y, Shahsavani A (2024). Abnormal frequency of the memory B cell subsets and plasmablasts in patients with congenital severe hemophilia A: correlation with “Inhibitor” formation. Blood Res.

